# Assessment of lower urinary tract symptoms 6 weeks after delivery and the relationship of pelvic floor muscle function

**DOI:** 10.3389/fgwh.2024.1416429

**Published:** 2024-12-02

**Authors:** Lei Zhang, Xiaoxiao Wang, Xinnan Hou, Xinrong Zhuang, Yu Wang, Xiaoqing Wang, Ye Lu

**Affiliations:** ^1^ Department of Obstetrics and Gynecology, Peking University First Hospital, Beijing, China; ^2^ Department of Obstetrics and Gynecology, The Affiliated Hospital of Chengde Medical University, Chengde, China

**Keywords:** lower urinary tract symptoms (LUTS), prevalence, potential risk factors, bother, pelvic floor muscle function

## Abstract

**Background:**

Studies on lower urinary tract symptoms (LUTS) in women, especially in relation to different modes of delivery, are limited. The relationship between the emergence of LUTS and the decline of pelvic muscle function after childbirth remains uncertain.

**Study design:**

This observational study was carried out at the Peking University First Hospital over a time span of 2019–2022. A total of 2,462 women were recruited and surveyed 6 weeks after delivery, utilizing questionnaires for data collection. Additionally, gynecological physical examinations and pelvic floor muscle screenings were conducted as part of the study. To assess individual LUTS and the level of discomfort caused by these symptoms, a modified Chinese version of the Bristol Female Lower Urinary Tract Symptoms questionnaire was employed. Data analysis methods such as descriptive statistics, *χ*^2^ tests, one-way ANOVAs, and multivariate logistic regressions were used to thoroughly examine the collected data.

**Results:**

A high prevalence of LUTS was observed in the study participants, with 70.6% experiencing any symptoms. Storage symptoms were reported by 65.4%, while voiding symptoms were reported by 23.0%. Nocturia was the most commonly reported symptom (35.4%), followed by frequency (25.6%) and urgency (25.3%). Stress urinary incontinence (SUI) was reported by 20.8% of women. Interestingly, nocturia and frequency were generally perceived as less troublesome, with only a minority rating them as problematic. In contrast, urinary incontinence (UI) was frequently reported as highly bothersome, with SUI and urge urinary incontinence (UUI) accounting for significant proportions. Vaginal delivery (VD) and forceps delivery (FD) were identified as significant predictors of LUTS, with statistical significance observed (*P* < 0.05). Specifically, women who underwent VD, particularly FD, exhibited lower surface electromyography (sEMG) activity compared to those who had cesarean section (CS), both in terms of resting baseline and contraction amplitude (*P* < 0.001).

**Conclusions:**

Over half of the examined women exhibited LUTS 6 weeks postpartum, with the most common symptoms being nocturia, frequency, urgency, and SUI. Straining and urinary incontinence were commonly reported as significantly uncomfortable, particularly severe in cases of UI. Additionally, vaginal delivery methods, especially those involving the use of forceps (FD), seemed to be more likely to cause pelvic floor muscle or nerve damage, making it the key predictor of storage-related LUTS.

## 1 Introduction

In recent years, lower urinary tract symptoms (LUTS) have garnered significant attention, primarily due to their high prevalence and the adverse impact they have on individuals’ health-related quality of life (1). This heightened awareness has led to a growing concern about LUTS, which are particularly common in women and can negatively affect their quality of life. However, most women do not seek treatment for these symptoms (2, 3).

While several effective treatment options exist for LUTS, there is a limited amount of evidence guiding optimal strategies for bladder health promotion and LUTS prevention. Most primary prevention trials focus solely on a single symptom, with limited research addressing other LUTS such as voiding dysfunction or pain (4–7).

Evidence does suggest that pelvic floor exercise programs can be beneficial for the prevention of urinary incontinence (UI) during pregnancy. However, the outcomes for other LUTS remain unknown. Pregnancy and vaginal birth are recognized as risk factors for UI due to the potential damage to pelvic floor musculature and nerves (8–11).

The postpartum period is a crucial time for women as they recover and adjust to their new roles. Traditional practices are observed to aid in this recovery and prevent future health issues (12). It is during this time that measures can be taken to prevent and promote UI. However, there is a dearth of data on LUTS in this population. By examining the effects of labor management practices on urological outcomes during this critical period, researchers can provide valuable insights to improve maternity care policies and practices.

The aim of this study is to provide estimates of the prevalence and potential risk factors for LUTS in women six weeks after different modes of delivery. Furthermore, we aim to assess the burden these symptoms have on women and explore the relationship between pelvic floor function and various types of LUTS.

## 2 Material and methods

### 2.1 Study design and participants

Starting from January 2019, we initiated data collection from women who underwent postpartum reviews at six weeks after delivery at the outpatient clinics of Peking University First Hospital. We will inquire about their urinary incontinence status prior to pregnancy. Women who do not experience urinary incontinence are invited to participate in this research. We have excluded women who:(a) Have been medically diagnosed with cognitive impairments, rendering them unable to understand the questions; (b) Have had a parity of three or more; (c) Have experienced multiple pregnancies; (d) Are diagnosed with pelvic organ prolapse at stage 2 or higher; (e) Have a history of anti-incontinence or pelvic organ prolapse surgery, or a history of urinary surgery; (f) Have a diagnosis of a malignant tumor; (g) Are currently experiencing a urinary tract infection; (h) Do not wish to participate.

Questionnaires were used to investigate these women, and they also underwent a comprehensive gynecological examination and a pelvic floor muscle screening (PFM screening). We have obtained approval from the ethics committee and obtained written consent from all participants. We calculated the sample size based on our previous national study (13). Our aim was to survey at least three different delivery modes. To detect a 55.5% prevalence of lower urinary tract symptoms (LUTS) with a 5% estimated error and a 95% confidence interval (CI), a minimum sample size was determined. Taking into account a 20% refusal rate, a total of 1,367 participants would be required, as calculated by the following formula:

$$N\;=\;Z_{\alpha /2}^{2}\;\times \;(\pi \;\times \;(1\;-\;\pi ))\;/\;\delta^{2}\;\times \;3\;\times \;(1\;+\;20\text{\%})\;=\;1.96^{2}\;\times \;(55.5\text{\%}\;\times \;(1\text{–}55.5\text{\%}))\;/\;0.05^{2}\;\times \;3\times \;(1\;+\;20\text{\%})\approx 1367.$$

### 2.2 measurements

We employed the Chinese version of the International Consultation on Incontinence Questionnaire–Female Lower Urinary Tract Symptoms (ICIQ-FLUTS) for our investigation (14). This questionnaire comprehensively assessed ten different types of LUTS, including nocturia, daytime frequency, urgency, urge urinary incontinence (UUI), stress urinary incontinence (SUI), other forms of incontinence, pain or burning sensation, hesitancy, straining, and intermittency.

Participants were asked to rate the frequency and degree of bother associated with each LUTS. For the prevalence of LUTS, responses indicating any experience other than “no” were considered. To evaluate the level of bother, a scale ranging from 0 (not bothered at all) to 10 (greatly bothered) was used. We further categorized the bother responses into three groups: minor (scores 1–4), moderate (scores 5–7), and severe (scores 8–10). In addition to the LUTS assessment, we also collected detailed information on sociodemographic background, reproductive factors, defecation habits, and any medical conditions that the participants may have. This comprehensive approach allowed us to gain a comprehensive understanding of the women's health status and their experiences with LUTS.

### 2.3 Diagnostic criteria

The definitions employed in this study align with the standards recommended by the 2002 guidelines of the International Continence Society (ICS) (15). Within our study, we defined nocturia as occurring when a woman experiences two or more micturitions during the night. Additionally, daytime frequency was categorized as voiding eight times or more per day. For those who reported urinary incontinence (UI) without symptoms of mixed UI (UUI) or stress UI (SUI), we classified them as having a different type of UI. As for cesarean sections (CS), if performed after cervical dilation of 1 cm or more during labor, it was categorized as CS during labor. Otherwise, it was defined as a CS not performed during labor.

### 2.4 Pelvic floor muscle screening (PFM screening)

During the study, all participants underwent a comprehensive gynecological examination, including a physical assessment and a screening for pelvic floor muscles (PFMs). To record the bioelectrical activity of the PFMs, we utilized equipment from Nanjing Medlander Company (China) and followed the Glazer protocols. The patients were positioned in a supine position with gently flexed hips and knees. Four monitoring electrodes were strategically placed: two on the iliac region and the other two on both sides of the hypogastric region. After inserting the vaginal electrode, participants were instructed to perform a series of contraction and relaxation movements in response to voice commands.

The surface electromyography (sEMG) signal parameters recorded included:.

1. A 60-second rest period (pre-baseline) to measure the average mean amplitude (in microvolts, μV) and mean amplitude variability (%).
2. Five phasic contractions of 2 s each, with a 2 s rest between each contraction, to capture the average peak amplitude (μV), time before peak (in seconds), and time after peak (in seconds).
3. Five tonic contractions lasting 10 s each, with a 10 s rest between each contraction, to measure the average mean amplitude (μV) and mean amplitude variability (%).
4. Another 60 s rest period (post-baseline) to reassess the average mean amplitude (μV) and mean amplitude variability (%).

### 2.5 Statistical analysis

To assess the prevalence differences between groups, we conducted *χ*^2^ tests. A comprehensive multivariable model was established to evaluate potential risk factors related to bothersome and individual lower urinary tract symptoms (LUTS). This model was refined until it reached its final version, estimating odds ratios and 95% confidence intervals. A statistically significant difference was determined by a two-sided *P* value of ≤0.05. Additionally, one-way ANOVA was applied with Bonferroni correction for further analysis. We utilized EpiData software for data entry and error detection purposes, while SPSS version 12.0 (IBM Corp., Armonk, NY, USA) was employed for the statistical analysis of our data.

## 3 Results

**Figure 1:**
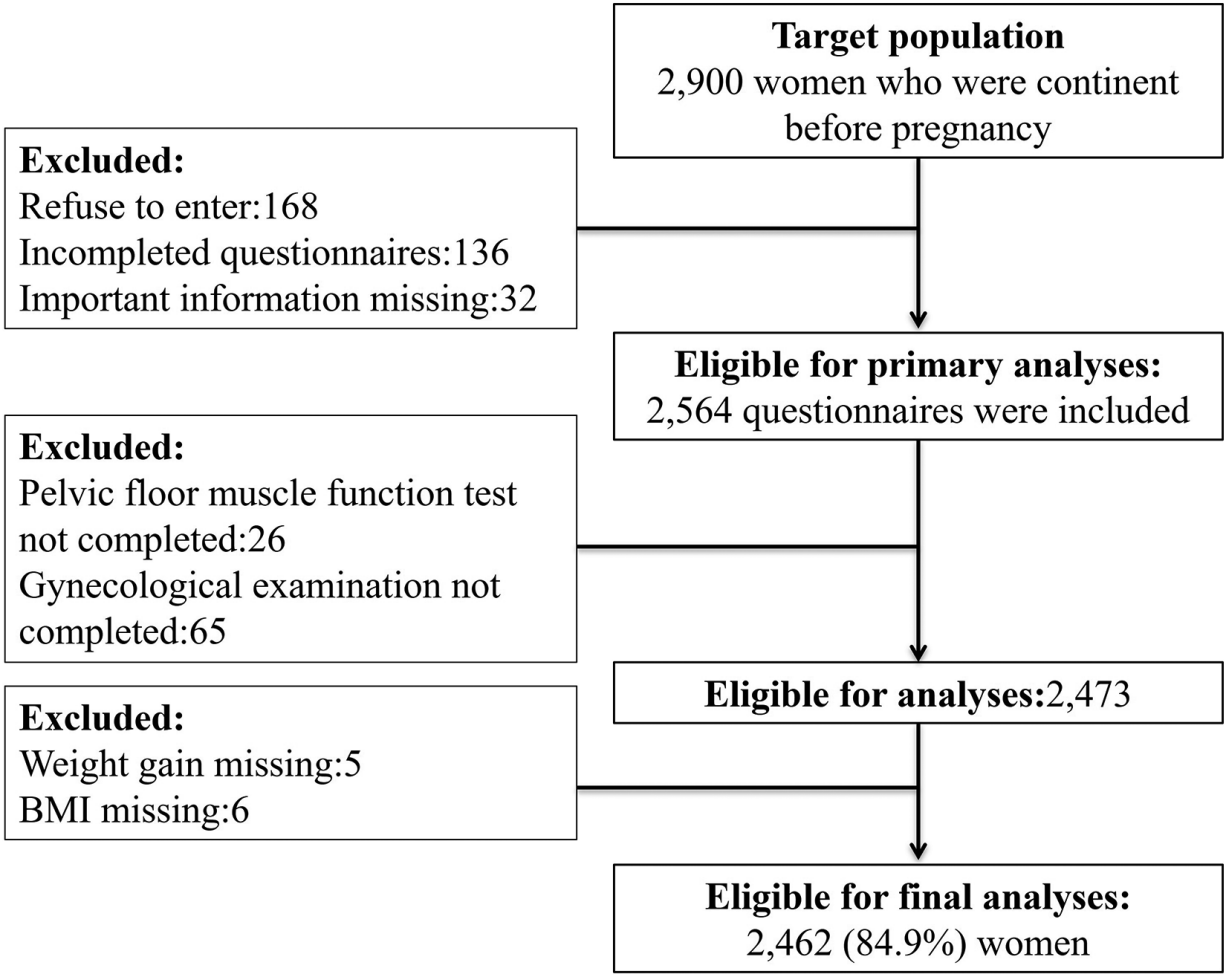
Study flowchart.

According to the flowchart (Figure 1), we began with 2900 participants at the baseline. After data collection, we were able to include 2,462 participants (84.9%) in our final data analysis. The age range of these participants spanned from 20 to 46 years, with a mean age of 32.4 years ± 3.8 years. In terms of delivery methods, 1,274 (51.7%) had vaginal deliveries (VDs), 692 (28.1%) underwent cesarean sections (CS) (not during labor), 236 (9.6%) had CSs (during labor), and 260 (10.6%) had forceps deliveries (FDs). Additionally, 525 women (21.3%) had a history of delivery. Table 1 provides a detailed overview of the sociodemographic characteristics of our participants.

**Table 1:** Characteristics of the 2,462 subjects interviewed.

Characteristic	*N*=2,462
Age (years), mean (SD), range	32.37 (3.8) (20–46)
Age (years), *N* (%)	
20–29	522 (21.2)
30–39	1,841 (74.8)
40–49	99 (4.0)
Delivery history, *N* (%)	525 (21.3)
Vaginal delivery	296 (56.4)
Cesarean section	201 (38.3)
Forceps delivery	18 (3.4)
Induction of labor	10 (1.9)
Delivery mode, *N* (%)	
Vaginal delivery	1,274 (51.7)
Cesarean section (not in labor)	692 (28.1)
Cesarean section (in labor)	236 (9.6)
Forceps delivery	260 (10.6)
Fetal weight (kg), *N* (%)	
<2.5	176 (7.1)
2.5–3.0	482 (19.6)
3.0–3.5	1,128 (45.8)
3.5–4.0	595 (24.2)
≥4.0	81 (3.3)
Perineal laceration, *N* (%)	785 (51.2)^a^
No	113 (14.4)
1	583 (74.3)
2	86 (11.0)
3	2 (0.3)
4	1 (0.1)
Lateral episiotomy, *N* (%)	
Yes	852 (55.5)^b^
No	682 (44.5)
Stage of labor (min), mean (SD)	
The first labor	575.5 (335.3)
The second labor	42.7 (43.3)
The third labor	8.1 (7.6)
Progestational BMI (Kg/m^2^), *N* (%)	
Underweight (<18.5)	258 (10.5)
Normal (18.5–23.9)	1,634 (66.4)
Overweight (24–27.9)	462 (18.8)
Obese (≥28)	108 (4.4)
Later trimester BMI (Kg/m^2^), *N* (%)	
Underweight (<18.5)	0 (0)
Normal (18.5–23.9)	464 (18.8)
Overweight (24–27.9)	1,197 (48.6)
Obese (≥28)	801 (32.5)
Weight gain (Kg), *N* (%)	
<10 kg	390 (15.8)
10–15 kg	1,194 (48.5)
15–20 kg	644 (26.2)
≥20 kg	234 (9.5)
Job, *N* (%)	
Physica labor	230 (9.3)
Mental labor	2,232 (90.7)
Race, *N* (%)	
Han	2,309 (93.8)
Minority	153 (6.2)
Pelivc surgery history	
Yes	429 (17.4)
No	2,033 (82.6)
Gynecological complications	
Yes	187 (7.6)
No	2,275 (92.4)
Complications	
No	1,180 (47.9)
GDM	632 (25.7)
Hypothyroidism	283 (11.5)
Gestational hypertension	190 (7.7)
Mild preeclampsia	85 (3.5)
Severe preeclampsia	67 (2.7)
Chronic hypertension	64 (2.3)
Connective tissue diseases	90 (3.7)
Hyperthyroidism	79 (3.2)
Dibetes	78 (3.2)
Others	484 (19.7)
Smoking	
Yes	10 (0.4)
No	2,452 (99.6)
Alcohol history	
Yes	4 (0.2)
No	2,458 (99.8)

Pelvic surgery history included: Oophorocystectomy, appendectomy, cesarean section, myomectomy and salpingectomy.

^a^ The women had perineal laceration/women had vaginal delivery.

^b^ The women had Lateral episiotomy/women had vaginal delivery.

### 3.1 Prevalence of lower urinary tract symptoms

The frequencies of LUTS are detailed in Table 2. The overall prevalence of any LUTS was 70.6%, which significantly varied with different delivery modes (*P* < 0.001). Storage symptoms were more frequently reported than voiding symptoms (65.4% vs. 23.0%).Storage LUTS were more prevalent in women who had vaginal deliveries (VDs) and forceps deliveries (FDs) compared to those who had cesarean section (CS) deliveries. Specifically, 70.3% of women with VDs and 73.5% of women with FDs experienced storage LUTS, compared to 53.4% and 57.4% respectively for CS deliveries, whether in labor or not. Nocturia, the most common symptom (35.4%), also varied significantly with different delivery modes (*P* < 0.001), with higher rates reported in women with VDs (34.8%), FDs (46.5%), and CS deliveries during labor (38.1%) and CS deliveries not in labor (31.5%). Other symptoms such as urgency (25.3%) and stress urinary incontinence (SUI) (20.8%) also differed significantly with different delivery modes (*P* < 0.001). In contrast, straining, the least common symptom (6.1%), showed a significant variation with different delivery modes (*P* = 0.01), as shown in Table 2.

**Table 2:** Prevalence of individual LUTS by age and different mode of delivery and delivery history.

Symptoms *N* (%)		Delivery mode					Delivery history				Age			
	Total	VD	Forceps delivery	CS (in labor)	CS (not in labor)	*P*-value^b^	VD history	Forceps delivery history	CS history	*P*-value^b^	20–29	30–39	40–49	*P*-value^b^
	*N* = 2,462	*N* = 1,274	*N* = 260	*N* = 236	*N* = 692		*N* = 306	*N* = 18	*N* = 201		*N* = 522	*N* = 1,841	*N* = 99	
Nocturia	872 (35.4)	443 (34.8)	121 (46.5)	90 (38.1)	218 (31.5)		90 (29.4)	5 (27.8)	62 (30.8)		183 (35.1)	650 (35.3)	39 (39.4)	
≥2 times/night						*<0.001*				*0.923*				*0.696*
Frequency	631 (25.6)	322 (25.3)	72 (27.7)	66 (28.0)	171 (24.7)	*0.649*	65 (21.2)	9 (50.0)	46 (22.9)	*0.019*	121 (35.1)	487 (26.5)	23 (23.2)	*0.2731*
Urgency	623 (25.3)	381 (29.9)	79 (30.4)	30 (12.7)	133 (19.2)	*<0.001*	88 (28.8)	3 (16.7)	40 (19.9)	*0.056*	141 (27.0)	458 (24.9)	24 (24.2)	*0.594*
Urinary incontinence														
Any UI	721 (26.6)	473 (37.1)	98 (37.7)	26 (11.0)	89 (12.9)	*<0.001*	149 (48.7)	7 (38.9)	52 (25.9)	*<0.001*	141 (27.0)	556 (30.2)	24 (24.2)	*0.196*
UUI	315 (12.8)	212 (16.6)	55 (21.2)	13 (5.5)	35 (5.1)	*<0.001*	55 (18.0)	0	17 (8.5)	*0.002*	64 (12.3)	243 (13.2)	8 (8.1)	*0.305*
SUI	512 (20.8)	341 (26.8)	64 (24.6)	18 (7.6)	89 (12.9)	*<0.001*	125 (40.8)	5 (27.8)	42 (20.9)	*<0.001*	80 (15.3)	412 (22.4)	20 (20.2)	*0.002*
MUI	198 (8.0)	136 (10.7)	30 (11.5)	9 (3.8)	23 (3.3)	*<0.001*	46 (15.0)	0	13 (6.5)	*0.004*	32 (6.1)	159 (8.6)	7 (7.1)	*0.166*
Other UI										*<0.001*				*0.006*
Any involuntary^a^	79 (3.2)	45 (3.5)	15 (5.8)	5 (2.1)	14 (2.0)	*0.019*	15 (4.9)	0	7 (3.5)	*0.491*	24 (4.6)	52 (2.8)	3 (3.0)	*0.127*
Nocturnal enuresis	14 (0.6)	7 (0.5)	0	3 (1.3)	4 (0.6)	*0.314*	1 (0.3)	0	4 (2.0)	*0.154*	6 (1.1)	7 (0.4)	1 (1.0)	*0.100*
Pain/Burning	274 (11.1)	152 (11.9)	39 (15.0)	15 (6.4)	68 (9.8)	*0.01*	17 (5.6)	2 (11.1)	19 (9.5)	*0.206*	74 (14.1)	197 (10.7)	3 (3.0)	*0.003*
Hesitancy	311 (12.6)	164 (12.9)	40 (15.4)	27 (10.4)	80 (11.6)	*0.411*	34 (11.1)	2 (11.1)	22 (10.9)	*0.998*	74 (14.1)	229 (12.4)	8 (8.1)	*0.218*
Straining	150 (6.1)	82 (6.4)	26 (10.0)	12 (5.1)	30 (4.3)	*0.01*	14 (4.6)	1 (5.6)	11 (5.5)	*0.895*	37 (7.1)	113 (6.1)	0	*0.026*
Intermittency	252 (10.2)	136 (10.7)	37 (14.2)	22 (9.3)	57 (8.2)	*0.046*	26 (8.5)	1 (5.6)	17 (8.5)	*0.908*	58 (11.1)	189 (10.3)	5 (5.1)	*0.189*
Storage symptoms	1,610 (65.4)	896 (70.3)	191 (73.5)	126 (53.4)	397 (57.4)	*<0.001*	217 (70.9)	12 (66.7)	115 (57.2)	*0.006*	339 (64.9)	1,204 (65.4)	67 (67.7)	*0.872*
Voiding symptoms	566 (23.0)	305 (23.9)	74 (28.5)	43 (18.2)	144 (20.8)	*0.019*	57 (18.6)	4 (22.2)	39 (19.4)	*0.919*	138 (26.4)	416 (22.6)	12 (12.1)	*0.006*
Any LUTS	1,738 (70.6)	950 (74.6)	203 (78.1)	140 (59.3)	445 (64.3)	*<0.001*	222 (72.5)	13 (72.2)	130 (64.7)	*0.164*	370 (70.8)	1,298 (70.5)	70 (70.7)	*0.986*

The italics value represents the *P* value.

^a^ “Any involuntary” represents women who reported leaking for no reason or any involuntary loss of urine.

^b^ Pearson's chi-square test was used to compare the prevalence differences of individual LUTS by age and different mode of delivery and delivery histories.

**Figure 2:**
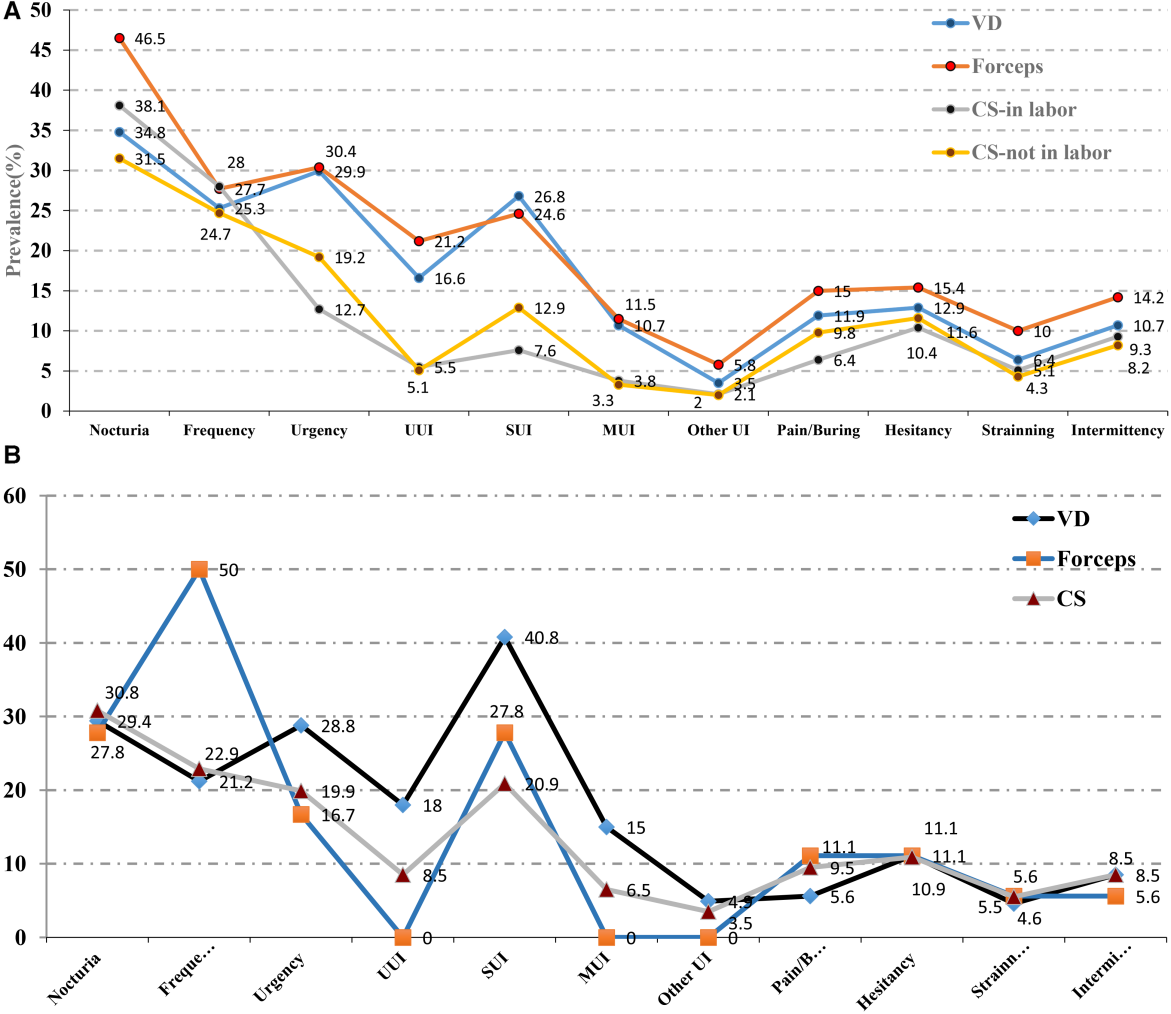
**(A)** The prevalence of different kind of LUTS between different delivery modes. **(B)** The prevalence of different kind of LUTS between different delivery histories.

Furthermore, women with a history of VD or FD were found to be more susceptible to storage LUTS such as frequency (*P* = 0.019) and UI (*P* < 0.001) compared to those with a history of CS, as shown in Table 2; Figure 2. However, there was no notable difference in the incidence of voiding symptoms between the groups. Additionally, young patients were at a higher risk for voiding symptoms like pain/burning (*P* = 0.003), while SUI was most frequently reported in women aged between 30 and 39 years old (22.4%).

### 3.2 Symptom-specific bother due to lower urinary tract symptoms

**Figure 3:**
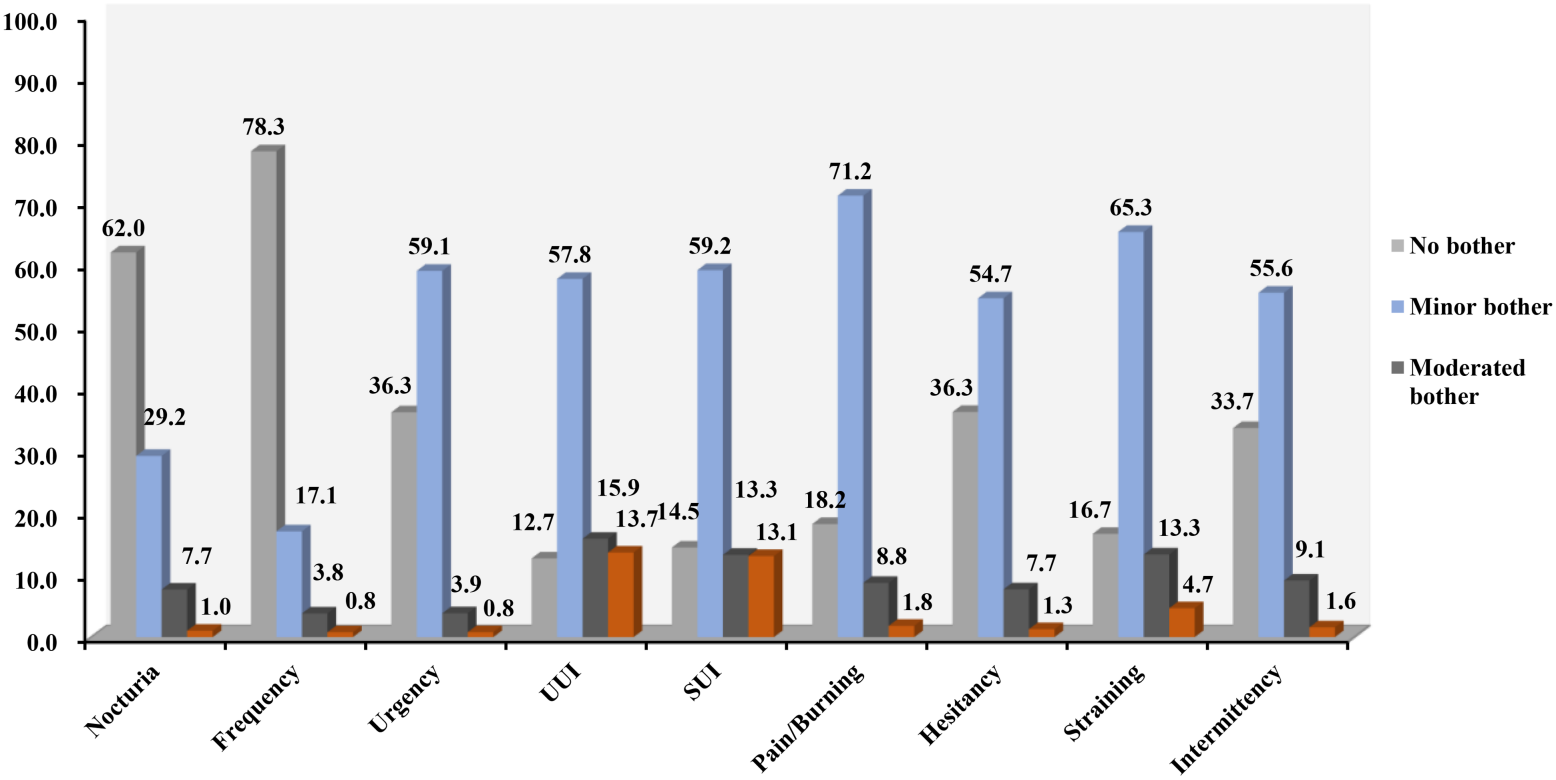
Symptom-specific bother and extent of individual LUTS. The bother associated with each symptom was evaluated using a scale ranging from 0 (not bothered at all) to 10 (greatly bothered). Bother was also defined by dividing the Likert responses into three groups: “minor” with a scale ranging from 1 to 4; “moderate” ranging from 5 to 7; and “severe” ranging from 8 to 10.

The study participants' level of discomfort regarding various urinary symptoms has been summarized in Table 3 and presented in Figure 3. It is notable that only 6.0%–20.2% of those with bothersome lower urinary tract symptoms (LUTS) reported a moderate to severe impact on their quality of life, with 1.3%–15.6% experiencing severe discomfort. Among the reported symptoms, UUI (87.3%) and SUI (85.5%) were the most prevalent and bothersome, respectively. However, it is interesting to note that symptoms like pain/burning (81.8%), while highly prevalent, were not the most bothersome, with only 10.7% rating it as moderately or severely bothersome. Among the symptoms, UUI was the most likely to cause severe (15.6%) or moderate to severe bother (18.1%), closely followed by SUI (15.3% and 15.5%, respectively). In contrast, nocturia, despite being less bothersome, was more likely to result in moderate to severe bother (38.0% and 20.2%). Frequency, which is a storage symptom, was reported to cause moderate or severe bother in 21.5% and 17.6% of cases, making it the least frequently bothersome among the storage symptoms.

**Table 3:** Self-reported bother related to individual LUTS.

*N* (%)	Symptoms								
	Nocturia	Frequency	Urgency	UUI^a^	SUI^a^	Pain/Burning	Hesitancy	Straining	Intermittency
0	541 (62.0)^b^	491 (77.8)^b^	226 (36.3)^b^	40 (12.7)^b^	74 (14.4)^b^	50 (18.2)^b^	113 (36.3)^b^	25 (16.7)^b^	85 (33.7)^b^
1	119 (36.0)^c^	55 (40.4)^c^	128 (32.2)^c^	87 (31.6)^c^	157 (35.8)^c^	106 (38.7)^c^	87 (43.9)^c^	41 (32.8)^c^	66 (39.5)^c^
2	96 (29.0)	47 (34.6)	87 (21.9)	45 (16.4)	71 (16.2)	53 (23.7)	43 (21.7)	29 (23.2)	36 (21.6)
3	52 (15.7)	14 (10.3)	71 (17.9)	36 (13.1)	52 (11.9)	26 (11.6)	32 (16.2)	28 (22.4)	28 (17.1)
4	8 (2.4)	5 (3.7)	24 (6.0)	16 (5.8)	27 (6.2)	13 (5.8)	11 (5.6)	3 (2.4)	11 (6.6)
5	30 (9.1)	10 (7.4)	34 (8.6)	31 (11.3)	39 (8.9)	13 (5.8)	16 (8.1)	16 (12.8)	11 (6.6)
6	8 (2.4)	3 (2.2)	14 (3.5)	8 (2.9)	18 (4.1)	3 (1.3)	7 (3.5)	1 (0.5)	9 (5.4)
7	9 (2.7)	1 (0.7)	8 (2.0)	9 (3.3)	7 (1.6)	5 (2.2)	0 ()	1 (0.5)	2 (1.2)
8	3 (0.9)	3 (2.2)	14 (3.5)	17 (6.2)	20 (4.6)	3 (1.3)	2 (1.0)	4 (3.2)	2 (1.2)
9	2 (0.6)	1 (0.7)	7 (1.8)	4 (1.5)	13 (3.0)	1 (0.4)	0	0	0
10	4 (1.2)	1 (0.7)	10 (2.5)	22 (8)	34 (7.8)	1 (0.4)	0	2 (1.6)	2 (1.2)
Any bother	331	136	397	275	438	224	198	125	167
Moderated to severe bother	67 (20.2)	24 (17.6)	24 (6.0)	50 (18.1)	68 (15.5)	24 (10.7)	24 (12.1)	20 (16.0)	23 (13.8)
Any severe bother	9 (2.7)	5 (3.7)	5 (1.3)	43 (15.6)	67 (15.3)	5 (2.2)	4 (2.0)	7 (5.6)	4 (2.4)
Any LUTS	872	631	623	315	512	274	311	150	252
Bother rate	38.0	21.5	63.7	87.3	85.5	81.8	63.7	83.3	66.3

^a^ “SUI” and “UUI” included the participants with MUI.

^b^ The number of every LUTS/any LUTS.

^c^ The number of every bother LUTS the number of every LUTS which cause bother. Any bother: the participants rated that they were bothered by individual LUTS.

When considering voiding symptoms, straining was reported as the most common bothersome symptom (83.3%). Interestingly, straining and intermittency were more likely to cause moderate or severe bother, at 16.0% and 13.8%, respectively. This information is comprehensively presented in Table 3; Figure 3.

### 3.3 Potential risk factors for lower urinary tract symptoms

The logistic regression analysis has revealed the risk factors for lower urinary tract symptoms (LUTS), as presented in Tables 4–6. The data indicates that both vaginal deliveries (VDs) and forceps deliveries (FDs) significantly increase the odds of experiencing various types of LUTS. Women who had VDs and FDs were found to be more susceptible to any LUTS and bothersome LUTS compared to those who had cesarean sections (CS). Specifically, the odds ratios were notably high for severe and bothersome symptoms, ranging from 2.11-fold to 6.65-fold. Additionally, a history of VD or FD was identified as a strong predictor for moderate to severely bothersome LUTS, any severely bothersome LUTS, and urinary incontinence (UI). The presence of a perineal laceration was also found to increase the odds of experiencing any bothersome LUTS and stress urinary incontinence (SUI). However, voiding symptoms seemed to be less affected by the mode of delivery and delivery histories.

**Table 4:** Multivariate analyses of the associations of characteristics with the presence of bothersome LUTS.

		Any bother LUTS *N* = 1,045			Moderate to severe bother LUTS *N* = 256			Any severe bother LUTS *N* = 101		
	*N* (%)	*P*-value	Adjusted OR	95% CI	*P*-value	Adjusted OR	95% CI	*P*-value	Adjusted OR	95% CI
Age group (years)		*0.244*			*0.675*			*0.087*		
20–29 (ref.)	522 (21.2)									
30–39	1,841 (74.8)	*0.252*	1.13	(0.92, 1.40)	*0.733*	1.06	(0.76, 1.47)	*0.057*	1.74	(0.98, 3.08)
40–49	99 (4.0)	*0.463*	0.83	(0.51, 1.36)	*0.488*	0.71	(0.27, 1.87)	*0.523*	0.51	(0.07, 4.01)
Job, *N* (%)		*0.990*	1.00	(0.83, 1.21)	*0.536*	1.10	(0.82, 1.47)	*0.317*	1.25	(0.81, 1.92)
Physical labor	739 (30.0)									
Mental labor (ref.)	1,723 (70.0)									
Delivery mode, *N* (%)		***<0.001***			***<0.001***			***<0.001***		
Vaginal delivery	1,274 (51.7)	***<0.001***	**2.11**	**(1.69, 2.63)**	***<0.001***	**3.17**	**(2.03, 4.92)**	***<0.001***	**4.80**	**(2.22, 10.39)**
CS (not in labor) (ref.)	692 (28.1)									
CS (in labor)	236 (9.6)	*0.131*	0.76	(0.54, 1.08)	*0.416*	1.33	(0.67, 2.61)	*0.194*	2.06	(0.69, 6.15)
Forceps delivery	260 (10.6)	***<0.001***	**2.55**	**(1.86, 3.50)**	***<0.001***	**4.14**	**(2.42, 7.07)**	***<0.001***	**6.65**	**(2.76, 16.03)**
Mode of delivery history		*0.681*			*0.014*			*0.029*		
Spontaneous VD	306 (58.3)	*0.415*	1.41	(0.62, 3.24)	***0.004***	**6.83**	**(1.87, 24.93)**	***0.008***	**16.33**	**(2.05, 129.85)**
Forceps or vacuum	18 (3.43)	*0.825*	1.17	(0.29, 4.65)	***0.030***	**8.12**	**(1.22, 54.06)**	*0.127*	11.00	(0.51, 238.31)
Caesarean section (ref.)	201 (38.3)									
Fetal weight (kg)		*0.215*			*0.429*			*0.488*		
<2.5	176 (7.1)	*0.414*	0.83	(0.66, 1.30)	*0.944*	0.97	(0.41, 2.27)	*0.972*	0.98	(0.28, 3.46)
2.5–3.0 (ref.)	482 (19.6)									
3.0–3.5	1,128 (45.8)	*0.105*	0.83	(0.66, 1.04)	*0.308*	1.22	(0.83, 1.81)	*0.895*	0.96	(0.54, 1.71)
3.5–4.0	595 (24.2)	*0.779*	1.04	(0.80, 1.35)	*0.096*	1.44	(0.94, 2.22)	*0.545*	1.22	(0.65, 2.29)
≥4.0	81 (3.3)	*0.961*	0.99	(0.59,1.65)	*0.190*	1.70	(0.77, 3.77)	*0.124*	2.30	(0.80, 6.65)
BMI before pregnant (Kg/m^2^)		*0.379*			*0.780*			*0.924*		
Underweight (<18.5)	258 (10.5)									
Normal (18.5–23.9)(ref.)	1,634 (66.4)	*0.838*	0.97	(0.69, 1.36)	*0.573*	0.86	(0.50, 1.47)	*0.773*	0.89	(0.39, 2.02)
Overweight (24–27.9)	462 (18.8)	*0.362*	0.84	(0.58, 1.65)	*0.481*	0.81	(0.45, 1.46)	*0.922*	0.96	(0.39, 2.35)
Obese (≥28)	108 (4.4)									
Late trimester BMI (Kg/m^2^)		*0.644*			*0.094*			*0.206*		
Normal (18.5–23.9) (ref.)	464 (18.8)									
Overweight (24–27.9)	1,197 (48.6)	*0.685*	1.17	(0.56, 2.43)	*0.104*	0.43	(0.16, 1.19)	*0.236*	0.43	(0.11, 1.73)
Obese (≥28)	801 (32.5)	*0.462*	1.35	(0.60, 3.04)	*0.571*	0.72	(0.24, 2.21)	*0.882*	0.90	(0.19, 4.23)
Weight gain (Kg)		*0.613*			*0.979*			*0.191*		
<10 kg (ref.)	390 (15.8)									
10–15 kg	1,194 (48.5)	*0.237*	0.85	(0.65, 1.11)	*0.844*	0.96	(0.62, 1.49)	*0.221*	0.68	(0.37, 1.26)
15–20 kg	644 (26.2)	*0.337*	0.86	(0.64, 3.0)	*0.690*	0.90	(0.55, 1.49)	*0.165*	0.61	(0.30, 1.23)
≥20 kg	234 (9.5)	*0.231*	0.79	(0.53, 1.17)	*0.805*	0.92	(0.50,1.73)	***0.034***	**0.31**	**(0.11, 0.92)**
Perineal laceration		***0.005***	**1.90**	**(1.21, 2.98)**	*0.100*	1.89	(0.89, 4.05)	*0.196*	2.11	(0.68, 6.51)
No (ref.)	113 (14.4)									
Yes	672 (85.8)									
Comlication		*0.515*	0.94	(0.79, 1.12)	*0.785*	1.04	(0.79, 1.37)	*0.773*	1.06	(0.70, 1.62)
No (ref.)	1,180 (47.9)									
Yes	1,282 (52.1)									
Pelvic Surgery history		*0.359*	1.12	(0.88, 1.42)	*0.833*	0.96	(0.63, 1.46)	*0.847*	1.06	(0.70, 1.62)
No (ref.)	2,033 (82.6)									
Yes	429 (17.4)									
Diabetes		*0.752*	0.88	(0.40, 1.92)	*0.821*	1.15	(0.34, 3.96)	*0.839*	0.81	(0.11, 6.26)
Nonexistent (ref.)	2,433 (98.8)									
Present	29 (1.2)									

CI, confidence interval; LUTS, lower urinary tract symptoms; OR, odds ratio. The adjusted odds ratios with 95% confidence intervals are presented. All of the factors, except for mode of delivery history and perineal laceration, were adjusted in the multivariable model. For mode of delivery history and perineal laceration, the other factors were adjusted. Diabetes included defined participants with hospital diagnosis. The bold values represent numbers with statistical differences. Italics represent *P*-values.

**Table 5:** Multivariate analyses of the associations of characteristics with the presence of storage, voiding and incontinence LUTS.

		Storage symptoms *N* = 1,610			Voiding symptoms *N* = 566			Any UI *N* = 721		
	*N* (%)	*P*-value	Adjusted OR	95% CI	*P*-value	Adjusted OR	95% CI	*P*-value	Adjusted OR	95% CI
Age group (years)		*0.297*			***0.016***			*0.083*		
20–29 (ref.)	522 (21.2)									
30–39	1,841 (74.8)	*0.576*	1.07	(0.86, 1.33)	*0.058*	0.80	(0.63, 1.01)	***0.026***	**1.30**	**(1.03, 1.64)**
40–49	99 (4.0)	*0.119*	1.48	(0.90, 2.43)	***0.009***	**0.40**	**(0.21, 0.80)**	*0.524*	1.19	(0.69, 2.05)
Job, *N* (%)		*0.347*	1.10	(0.90, 1.33)	*0.268*	1.13	(0.91, 1.39)	*0.687*	0.96	(0.78, 1.18)
Physical labor	739 (30.0)									
Mental labor (ref.)	1,723 (70.0)									
Delivery mode, *N* (%)		***<0.001***			*0.175*			***<0.001***		
Vaginal delivery	1,274 (51.7)	***<0.001***	**1.84**	**(1.48, 2.29)**	*0.806*	1.03	(0.80, 1.33)	***<0.001***	**2.79**	**(2.16, 3.60)**
CS (not in labor) (ref.)	692 (28.1)									
CS（in labor）	236 (9.6)	*0.267*	0.84	(0.61, 1.15)	*0.218*	0.77	(0.52, 1.16)	***0.002***	**0.47**	**(0.28, 0.76)**
Forceps delivery	260 (10.6)	***<0.001***	**2.18**	**(1.55, 3.06)**	*0.659*	1.28	(0.90, 1.83)	***<0.001***	**2.89**	**(2.04, 4.09)**
Mode of delivery history		*0.131*			*0.805*			*0.040*		
Spontaneous VD	306 (58.3)	*0.056*	2.49	(0.98, 6.31)	*0.630*	1.28	(0.47, 3.47)	***0.020***	**2.71**	**(1.17, 6.28)**
Forceps or vacuum	18 (3.43)	*0.494*	1.67	(0.38, 7.27)	*0.518*	1.75	(0.32, 9.48)	*0.597*	1.47	(0.35, 6.10)
Caesarean section (ref.)	201 (38.3)									
Fetal weight (kg)		*0.769*			*0.434*			*0.334*		
<2.5	176 (7.1)	*0.781*	0.94	(0.61, 1.45)	*0.591*	1.15	(0.69, 1.93)	*0.264*	1.32	(0.81, 2.16)
2.5–3.0 (ref.)	482 (19.6)									
3.0–3.5	1,128 (45.8)	*0.559*	1.07	(0.85, 1.36)	*0.095*	1.26	(0.96, 1.67)	*0.553*	1.08	(0.84, 1.40)
3.5–4.0	595 (24.2)	*0.254*	1.17	(0.89, 1.53)	*0.088*	1.31	(0.96, 1.79)	*0.069*	1.31	(0.98, 1.74)
≥4.0	81 (3.3)	*0.925*	1.03	(0.61, 1.71)	*0.935*	1.03	(0.54, 1.95)	*0.436*	1.25	(0.71, 2.20)
BMI before pregnant (Kg/m^2^)		*0.289*			*0.029*			*0.561*		
Underweight (<18.5)	258 (10.5)									
Normal (18.5–23.9)(ref.)	1,634 (66.4)	*0.630*	0.92	(0.64, 1.32)	*0.459*	1.16	(0.79, 1.71)	*0.344*	1.20	(0.82, 1.76)
Overweight (24–27.9)	462 (18.8)									
Obese (≥28)	108 (4.4)	*0.232*	0.79	(0.53, 1.17)	*0.419*	0.84	(0.54, 1.29)	*0.283*	1.26	(0.83, 1.90)
Late trimester BMI (Kg/m^2^)		*0.500*			*0.549*			*0.631*		
Normal (18.5–23.9) (ref.)	464 (18.8)									
Overweight (24–27.9)	1,197 (48.6)	*0.566*	1.25	(0.58, 2.69)	*0.819*	1.10	(0.48, 2.51)	*0.587*	1.27	(0.54, 2.98)
Obese (≥28)	801 (32.5)	*0.323*	1.53	(0.66, 3.56)	*0.769*	0.87	(0.35, 2.17)	*0.401*	1.49	(0.59, 3.78)
Weight gain (Kg)		*0.412*			*0.056*			*0.171*		
<10 kg (ref.)	390 (15.8)									
10–15 kg	1,194 (48.5)	*0.100*	0.79	(0.60, 1.05)	*0.069*	0.76	(0.56, 1.02)	*0.192*	0.82	(0.62, 1.10)
15–20 kg	644 (26.2)	*0.336*	0.86	(0.63, 1.17)	*0.176*	0.79	(0.56, 1.11)	*0.930*	1.02	(0.73, 1.41)
≥20 kg	234 (9.5)	*0.329*	0.82	(0.55, 1.23)	*0.558*	1.14	(0.74, 1.76)	*0.247*	0.78	(0.51, 1.19)
Perineal laceration		*0.720*	1.09	(0.68, 1.74)	*0.673*	1.12	(0.66, 1.93)	***0.009***	**1.88**	**(1.17, 3.03)**
No (ref.)	113 (14.4)									
Yes	672 (85.8)									
Comlication		*0.677*	1.04	(0.87, 1.24)	*0.161*	0.87	(0.71, 1.06)	*0.975*	1.00	(0.82, 1.21)
No (ref.)	1,180 (47.9)									
Yes	1,282 (52.1)									
Pelvic Surgery history		*0.666*	0.95	(0.74, 1.21)	*0.486*	0.90	(0.68, 1.20)	*0.682*	1.06	(0.81, 1.38)
No (ref.)	2,033 (82.6)									
Yes	429 (17.4)									
Diabetes		*0.093*	2.20	(0.88, 5.54)	*0.082*	0.28	(0.07, 1.18)	*0.419*	1.40	(0.62, 3.15)
Nonexistent (ref.)	2,433 (98.8)									
Present	29 (1.2)									

All of the factors, except for mode of delivery history and perineal laceration, were adjusted in the multivariable model. For mode of delivery history and perineal laceration, the other factors were adjusted. The bold values represent numbers with statistical differences. Italics represent *P*-values.

**Table 6:** Multivariate analyses of the associations of characteristics with the presence of individual and Any LUTS.

	*N* (%)	Symptoms											
		UUI *N* = 315	SUI *N* = 512	MUI *N* = 198	Nocturia *N* = 872	Frequency *N* = 631	Urgency *N* = 623	Other UI *N* = 120	Pain/Burning *N* = 274	Hesitancy *N* = 311	Straining *N* = 150	Intermittency *N* = 252	Any LUTS *N* = 1,738
		Adjusted OR, 95% CI											
Age group (years)													
20–29 (ref.)	522 (21.2)												
30–39	1,841 (74.8)		**1.6** **(1.2, 2.2)**	**1.5** **(1.0, 2.3)**					**0.7** **(0.5, 1.0)**				
40–49	99 (4.0)								**0.2** **(0.1, 0.7)**				
Job, *N* (%)													
Physical labor	739 (30.0)												
Mental labor (ref.)	1,723 (70.0)												
Delivery mode, *N* (%)													
Vaginal delivery	1,274 (51.7)	**3.8** **(2.5, 5.7)**	**2.8** **(2.1, 3.8)**	**4.0** **(2.4, 6.6)**			**1.7** **(1.3, 2.2)**						**1.7** **(0.6, 1.1)**
CS (not in labor) (ref.)	692 (28.1)												
CS (in labor)	236 (9.6)		**0.5** **(0.3, 0.9)**		**1.4** **(1.0, 2.0)**		**0.6** **(0.4, 0.9)**		**0.5** **(0.3, 1.0)**				
Forceps delivery	260 (10.6)	**5.1** **(3.1, 8.4)**	**2.7** **(1.8, 4.0)**	**4.8** **(2.6, 8.8)**	**2.1** **(1.5, 2.9)**		**1.8** **(1.2, 2.5)**	**2.4** **(1.2, 4.6)**		**2.1** **(1.1, 3.7)**			**2.1** **(1.4, 2.9)**
Mode of delivery history													
Spontaneous VD	306 (58.3)												
Forceps or vacuum	18 (3.43)	–		–									
Caesarean section (ref.)	201 (38.3)												
Fetal weight (kg)													
<2.5	176 (7.1)	**1.9** **(1.0, 3.6)**											
2.5–3.0 (ref.)	482 (19.6)												
3.0–3.5	1,128 (45.8)												
3.5–4.0	595 (24.2)									**1.5 (1.0, 2.2)**			
≥4.0	81 (3.3)												
BMI before pregnant (Kg/m^2^)													
Underweight (<18.5)	258 (10.5)												
Normal (18.5–23.9)(ref.)	1,634 (66.4)												
Overweight (24–27.9)	462 (18.8)												
Obese (≥28)	108 (4.4)												
Late trimester BMI (Kg/m^2^)													
Normal (18.5–23.9) (ref.)	464 (18.8)												
Overweight (24–27.9)	1,197 (48.6)												
Obese (≥28)	801 (32.5)												
Weight gain (Kg)													
<10 kg (ref.)	390 (15.8)												
10–15 kg	1,194 (48.5)												
15–20 kg	644 (26.2)							**2.1** **(1.0, 4.4)**	**0.6** **(0.4, 0.9)**				
≥20 kg	234 (9.5)												
Perineal laceration	672 (85.8)		**2.3** **(1.3, 3.9)**										
Comlication	1,282 (52.1)												
Pelvic Surgery history	429 (17.4)												
Diabetes	29 (1.2)												

Except for the history of delivery mode and perineal laceration, all other factors were taken into account in the multivariable model adjustments. As there are numerous factors involved in the study, we have only presented the Adjusted Odds Ratio (OR) and 95% Confidence Interval (CI) for factors with a *P*-value less than 0.005. The bold values represent numbers with statistical differences.

### 3.4 Pelvic floor muscle function

There exists a noteworthy correlation between diverse delivery methods and Pelvic Floor Muscle (PFM) values (*P* < 0.001). A distinct pattern is evident in the sEMG parameters, with the group undergoing Cesarean Section (not during labor) exhibiting superior total scores (*P* < 0.001), pretest average mean amplitude (*P* < 0.001), flick contraction average peak amplitude (*P* < 0.001), tonic contraction average mean amplitude (CS vs. VD, *P* < 0.001; CS vs. FD, *P* = 0.002), tonic contraction mean amplitude variability, and posttesting average mean amplitude (all *P* < 0.001). Notably, women who had CS during labor also showed enhanced PFM function in comparison to VDs, as evident in the total score (*P* < 0.001) and various other sEMG parameters.

Forceps Deliveries (FDs) exhibit a more adverse impact on PFMs, with the amplitudes of flick and tonic contractions being notably lower in the FD group at 29.3 (14.2) and 19.1 (10.1) respectively. These amplitudes were significantly lower compared to other groups (*P* < 0.001). Additionally, there was a statistical difference in the total score (*P* = 0.001) between VDs and FDs. Interestingly, whether a woman underwent CS while in labor did not significantly affect the total score, flick contraction average peak amplitude, or posttesting average mean amplitude.

In total, 503 women had a history of delivery. A significant difference was observed in the total sEMG scores among different groups (*P* = 0.038). Specifically, women with a history of VD had lower flick contraction average peak amplitude (*P* = 0.015) and lower pretest and posttest average mean amplitudes (*P* = 0.001 and *P* = 0.009) compared to women with a history of CS delivery (Table 7). Our findings did not reveal any notable differences between women with or without lateral episiotomies or perineal lacerations in relation to PFM values.

**Table 7:** Pelvic muscle function and the relationship between different mode of delivery, delivery histories, lateral episiotomy and perineal laceration.

	Delivery mode				*P*-value	Delivery history			*P*-value	Lateral episiotomy		*P*-value	Perineal laceration			*P*-value
	CS (not in labor) *N* = 692	CS (in labor) *N* = 236	VD *N* = 1,274	FD *N* = 260		CS *N* = 201	VD *N* = 296	FD *N* = 18		No *N* = 682	Yes *N* = 852		No *N* = 113	I *N* = 583	II *N* = 86	
The total scroe	63.3 (14.3)	61.3 (14.7)	59.7 (16.2)	55.4 (16.8)	***<0.001***	63.1 (14.4)	59.4 (16.7)	63.3 (10.7)	***0.038***	60.0 (16.0)	58.2 (16.6)	*0.034*	59.8 (16.6)	60.4 (15.9)	58.3 (16.2)	*0.523*
Rest(pre-baseline) -average mean(μV)	7.3 (4.1)	7.4 (3.6)	5.8 (3.6)	5.3 (3.3)	***<0.001***	7.0 (4.3)	5.6 (3.2)	5.4 (2.6)	***<0.001***	5.8 (3.3)	5.7 (3.8)	*0.546*	5.5 (3.4)	5.9 (3.3)	5.4 (2.7)	*0.283*
Rest(pre-baseline) -variability (%)	0.2 (0.2)	0.2 (0.2)	0.2 (0.2)	0.2 (0.1)	*0.209*	0.2 (0.1)	0.2 (0.2)	0.1 (0.04)	*0.334*	0.2 (0.2)	0.2 (0.1)	*0.709*	0.2 (0.2)	0.2 (0.2)	0.2 (0.2)	*0.829*
Flick contractions -average peak (μV)	38.5 (15.3)	37.7 (17.7)	34.1 (16.3)	29.3 (14.2)	***<0.001***	38.1 (14.5)	34.0 (16.5)	41.1 (20.7)	***0.008***	34.1 (16.2)	32.6 (16.0)	***0.087***	34.3 (18.5)	34.7 (16.5)	30.8 (14.2)	*0.142*
Flick contractions -time before peak (s)	0.4 (0.2)	0.5 (1.3)	0.4 (0.3)	0.5 (0.37)	***0.041***	0.4 (0.2)	0.5 (0.3)	0.4 (0.1)	*0.469*	0.5 (0.3)	0.5 (0.3)	*0.515*	0.5 (0.3)	0.4 (0.3)	0.5 (0.3)	*0.675*
Flick contractions -time after peak (s)	0.5 (0.4)	0.6 (0.5)	0.6 (0.5)	0.7 (0.7)	***<0.001***	0.5 (0.3)	0.6 (0.6)	0.4 (0.1)	*0.068*	0.6 (0.5)	0.6 (0.6)	*0.281*	0.6 (0.5)	0.6 (0.5)	0.6 (0.5)	*0.810*
Tonic contractions -average mean (μV)	25.8 (11.6)	25.5 (12.3)	22.7 (12.2)	19.1 (10.0)	***<0.001***	24.7 (10.4)	22.4 (11.8)	26.2 (17.7)	*0.059*	22.6 (11.6)	21.8 (12.2)	*0.178*	24.5 (15.0)	23.0 (11.8)	20.9 (10.2)	*0.129*
Tonic contractions -variability (%)	0.2 (0.1)	0.2 (0.1)	0.3 (0.1)	0.3 (0.1)	***<0.001***	0.2 (0.1)	0.3 (0.1)	0.3 (0.1)	*0.088*	0.3 (0.1)	0.3 (0.1)	***0.033***	0.3 (0.1)	0.3 (0.1)	0.2 (0.1)	*0.697*
Rest(post-baseline) -average mean (μV)	6.9 (4.1)	7.1 (3.8)	5.6 (3.9)	5.1 (3.2)	***<0.001***	6.6 (4.1)	5.5 (3.2)	5.3 (2.5)	***0.006***	5.6 (3.3)	5.5 (4.1)	*0.523*	5.3 (3.0)	5.7 (3.4)	5.2 (2.7)	*0.293*
Rest(post-baseline) -variability (%)	0.2 (0.1)	0.2 (0.1)	0.2 (0.1)	0.1(0.1)	*0.244*	0.2(0.1)	0.1(0.1)	0.2(0.1)	*0.147*	0.2 (0.1)	0.2(0.1)	*0.861*	0.2(0.2)	0.2(0.1)	0.1(0.1)	*0.075*

## 4 Discussion

In previous reports, studies on lower urinary tract symptoms (LUTS) 6 weeks postpartum were limited, with most focusing primarily on the prevalence of urinary incontinence (UI) and neglecting voiding symptoms. It is crucial to recognize that 6 weeks postpartum is a crucial recovery period for women, during which preventing or addressing UI can significantly impact their well-being. However, there is a scarcity of data regarding LUTS in this population at this critical juncture. In our study, we observed a higher prevalence of LUTS compared to previous large population-based studies in adult women (13). We found that each individual LUTS caused distress, with storage symptoms being more likely to cause moderate or severe bother than voiding symptoms. Specifically, nocturia and frequency stood out, suggesting that these storage symptoms may be more closely related to pelvic floor muscle (PFM) damage. Unfortunately, we did not verify this hypothesis in our study. Interestingly, we discovered that symptoms and discomfort did not align in a straightforward manner. For instance, nocturia and frequency were rated as bothersome less frequently, yet they were primarily perceived as moderate to severe bother. This insight can be instrumental in identifying patients who require urgent medical intervention.

Our current study presents contrasting findings from our previous research. In our previous study, we noted that urgency was often associated with moderate or severe bother, whereas nocturia was typically perceived as a minor issue. Given that urinary urgency incontinence (UUI), stress urinary incontinence (SUI), and straining seem to have a more detrimental impact on patients’ quality of life, these should be prioritized for medical intervention.

The precise pathogenesis of LUTS remains incompletely understood; nevertheless, it is regarded as a multifaceted process. A broader understanding of LUTS reveals that potential risk factors encompass age, race, micturition habits, lifestyle factors, and particularly delivery histories. Childbirth frequently results in damage to the pelvic floor musculature, which is further confirmed by our findings. Multiple factors may amplify vulnerability to pelvic floor muscular trauma during childbirth, including advanced age at delivery, delivery mode, prolonged labor duration, and second-stage labor management practices such as exposure to medical interventions and duration of pushing (16). Accumulated pelvic floor muscular tears across pregnancies, the number of childbirth episodes, and associated interventions may contribute to an elevated risk of LUTS and compromised bladder health. We conducted a comprehensive evaluation of the relationship between any type of LUTS, individual bothersome LUTS, various delivery methods, and pelvic muscle function associated with delivery modes and histories. Both vaginal deliveries (VD) and fetal extractions (FD) were identified as strong predictors for various LUTS. Interestingly, women who underwent cesarean deliveries, regardless of the number of pregnancies, did not exhibit an increased likelihood of experiencing any type of LUTS. Women who had undergone either single or multiple VDs were found to have an increased risk of storage-related and bothersome LUTS, aligning with prospective studies focusing on reproductive histories and the progression of LUTS. Perineal lacerations may significantly impact several aspects of LUTS. Voiding symptoms, on the other hand, were less frequently associated with VDs. VDs can cause injuries to pelvic muscles, nerves, and connective tissue of the pelvic floor, ultimately leading to a negative impact on bladder control.

Overweight and obese women may experience improvements in lower urinary tract symptoms (LUTS), particularly urinary incontinence (UI) (17, 18). While weight gain during pregnancy may potentially contribute to an increase in UI both during and after pregnancy (19), scientific evidence to support this assertion is limited. One study has suggested that weight gain during pregnancy may not be a significant risk factor for LUTS (19). Our findings align with this, as we did not observe a notable impact of factors such as age, fetal weight, higher BMI, complications, or prior pelvic surgery on individual or bothersome LUTS.

There have been only a few studies exploring vaginal sEMG during the early postpartum period (20). Potential pelvic floor injuries, such as stretching of the pelvic floor muscles (PFMs), innervating nerves, and connective tissue, that may occur during vaginal delivery (VD) are known to play a significant role in pelvic floor pathologies. Among various factors, VD, particularly instrumental VD, has been found to be the most influential in causing changes to PFM parameters. Our findings are consistent with this observation. The resting baseline sEMG plays a crucial role in maintaining the correct anatomical position of the urethra. Although some studies suggest that this remains unaffected by pregnancy (21), we have confirmed that the mode of delivery, particularly different modes such as forceps or vacuum delivery, can affect the strength of PFMs, with recovery occurring naturally within 1–3 months post-delivery (22). Interestingly, compared to deliveries using forceps, vacuum deliveries appear to be less harmful to the pelvic floor musculature (23).

Furthermore, the number of VDs has also been linked to pelvic floor damage (24). Women with a history of surgical delivery (SD) tend to have lower contractile abilities (flick) and resting baseline sEMG levels. A prospective cohort study involving multiple women found that those who delivered all their children via cesarean section (CS) had stronger PFMs compared to women who had at least one vaginal non-forceps birth or those with at least one vaginal forceps-assisted delivery. We have observed similar results. It was also noted that the forceps group exhibited the most significant improvement in strength over time (25). It is worth mentioning that there is no evidence to suggest that routine episiotomy can protect the PFMs from damage.

While surface electromyography (sEMG) measures electrical activity from the recruitment of motor units rather than muscle strength itself, several studies suggest a strong correlation between the number of activated motor units and muscle strength (26). Pregnancy can lead to a reduction in pelvic floor muscle (PFM) strength and endurance due to compression, stretching, or tearing of nerves, muscles, and connective tissue (27). In general, as the number of activated motor units increases, muscle contractions also intensify. Therefore, electrical activity is proportional to the level of muscle strength developed, representing PFM contractions and relaxations (20, 21). Although our studies did not directly link LUTS to a decline in pelvic floor function, there is a significant correlation between the increased prevalence of LUTS, particularly storage symptoms, and vaginal deliveries (VDs), especially forceps deliveries (FDs). We believe that the alterations in bladder function during the storage period are closely linked to changes in postpartum pelvic floor muscle function. Further research is essential to validate this hypothesis.

Our research aims to provide better interventions and treatments for postpartum lower urinary tract symptoms. In previous studies, most of the focus has been on the treatment of postpartum urinary incontinence, with less research on the treatment of other lower urinary tract symptoms such as urinary frequency and urgency. Michele's study (28) provides a good idea for this field by studying the effect of drug treatment on mixed urinary incontinence. We speculate that Ospemifene may also have a very good therapeutic effect on patients with severe lower urinary tract symptoms. Whether pelvic floor muscle rehabilitation or pelvic floor muscle co-functional training can play a good role remains to be confirmed by larger, long-term prospective studies.

Our research possesses several notable advantages: (1) the extensive sample size we employed facilitates the conduct of comprehensive cross-sectional multifactor studies, providing a broad spectrum of data for analysis. (2) We achieved a low loss-to-follow-up rate due to the fact that the majority of our participants were women who delivered in our hospital, which simplified the process of collecting reliable clinical data. (3) We employed a professional questionnaire to assess urinary symptoms, which was carefully designed based on stringent criteria outlined in the 2002 ICS guidelines. This enabled us to make comparisons with the findings of other studies, providing a valuable comparative framework.

However, our study is not without its limitations: (1) our questionnaire did not encompass an exhaustive evaluation of all LUTS, potentially missing important aspects that could have contributed to a more comprehensive understanding of the phenomena we studied. (2) The observational design of our study is not an ideal method to determine risk factors accurately. Longitudinal studies are necessary to better understand the temporal nature and associations of risk factors, providing a more nuanced understanding of the relationships between variables. (3) The surface EMG results we obtained cannot directly reflect muscle strength, structure, or anatomical position. This is an area that requires further investigation to provide a more comprehensive assessment of the pelvic floor muscles and their role in LUTS.

## 5 Conclusions

This study was an observational investigation into lower urinary tract symptoms (LUTS) in women six weeks after childbirth, considering various modes of delivery. Among the participants, 70.6% reported experiencing LUTS, with nocturia, frequency, urgency, and stress urinary incontinence (SUI) being the most commonly reported symptoms. Notably, straining and UI were identified as the most troublesome symptoms, causing severe discomfort. Nocturia, frequency, urge urinary incontinence (UUI), and straining were also found to cause moderate to severe discomfort.The occurrence of any LUTS, individual symptoms, and bothersome symptoms may be influenced by multiple factors. In particular, vaginal deliveries (VDs), especially forceps deliveries (FDs), were identified as the strongest predictors, particularly for storage-related symptoms. This may be attributed to the potential for VDs and FDs to cause damage to pelvic floor muscles or nerves. Our findings revealed lower sEMG activity, including resting baseline and contraction amplitudes, in women who had undergone VDs, particularly FDs. Further research is warranted to explore the relationship between changes in bladder function during the storage period and alterations in postpartum pelvic floor function. Understanding these relationships may lead to improved treatment strategies and management of LUTS in postpartum women.

## Data Availability

The raw data supporting the conclusions of this article will be made available by the authors, without undue reservation.
